# Reconfigurable Parametric Amplifications of Spoof Surface Plasmons

**DOI:** 10.1002/advs.202100795

**Published:** 2021-07-04

**Authors:** Xinxin Gao, Jingjing Zhang, Yu Luo, Qian Ma, Guo Dong Bai, Hao Chi Zhang, Tie Jun Cui

**Affiliations:** ^1^ Institute of Electromagnetic Space Southeast University Nanjing 210096 China; ^2^ State Key Laboratory of Millimeter Waves Southeast University Nanjing 210096 China; ^3^ Center of Intelligent Metamaterials Pazhou Laboratory Guangzhou 510330 China; ^4^ School of Electrical and Electronic Engineering Nanyang Technological University Nanyang Avenue Singapore 639798 Singapore

**Keywords:** phase‐matching conditions, reconfigurable parametric amplifier, spoof surface plasmon polaritons

## Abstract

Next‐generation inter‐chip communication requires ultrafast ultra‐compact interconnects. Designer plasmonics offers a possible route towards this goal. Further development of the plasmonic technique to circuit applications requires the direct amplification of plasmonic signals on a compact platform. However, significant signal distortions and limited operational speeds prevent the application of traditional MOS‐based amplifiers to plasmonics. Up to day, the amplification of surface plasmons without phase distortion has remained a scientific challenge. In this work, the concept of parametric amplification (PA) is transplanted to the plasmonics and is realized experimentally an ultrathin reconfigurable PA using a spoof surface plasmon polariton (SSPP) waveguide integrated with tunable and nonlinear varactors. The measured parametric gain in the experiment can reach up to 9.14 dB within a short nonlinear propagation length, for example, six SSPP wavelengths, in excellent agreement with the theoretical prediction. By tuning the bias voltage of varactors, the phase‐matching condition can be precisely controlled over a broad frequency band, enabling the authors to realize the multi‐frequency PA of plasmonic signals. Measured phase responses confirm that the plasmonic parametric amplifier can significantly suppress the signal distortions as compared with the traditional MOS‐based amplifier, which is a property highly desired for ultrafast wireless communication systems and integrated circuits.

## Graphical abstract

A reconfigurable parametric amplifier (PA) is proposed using a spoof plasmonic waveguide integrated with varactors. By tuning the bias voltage of varactors, the phase‐matching condition can be precisely controlled over a broad frequency band, enabling the authors to realize the multi‐frequency PA of plasmonic signals. More importantly, the PA can significantly suppress signal distortions as compared with traditional MOS‐based amplifiers.

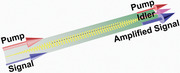

## 1 Introduction

We are in the era of information explosion facing an ever‐increasing demand for high‐speed information transfers. Communications based on the conventional electronic circuits are reaching the bandwidth limit due to unavoidable RC delays.^[^ ^1^ ^]^ Optical interconnects offer enormous bandwidth of up to tens of Tbps, but the inherent Abbe diffraction limit prevents high‐density photonic device integration, and the high coupling loss associated with the electro‐optic signal conversion further limits their applications.^[^ ^2^, ^3^, ^4^ ^]^ Spoof surface plasmon polariton (SSPP) was proposed as an alternative technology for inter‐chip communications, as it amasses the advantages of both electrical and optical interconnects.^[^ ^2^ ^]^ Suggested firstly by Pendry and coworkers in 2004, SSPPs are surface waves in the far‐infrared, terahertz, and microwave regions, mimicking the unique characteristics of surface plasmon polaritons (SPPs) via constructing corrugated metal surfaces.^[^ ^5^ ^]^

Similar to optical SPPs,^[^ ^6^, ^7^, ^8^, ^9^ ^]^ the SSPP modes can be squeezed far below the diffraction limit, allowing for highly confined electromagnetic energies.^[^ ^10^, ^11^, ^12^, ^44^ ^]^ On the other hand, the Ohmic loss can be significantly suppressed in the SSPP structures, since the electromagnetic fields are confined in the corrugations instead of penetrating the metal.^[^ ^13^ ^]^ Furthermore, since the artificial plasma frequency of SSPPs is dictated by the metal structure rather than the material itself, it offers us great freedom in designing the dispersion properties to meet the specific requirement.^[^ ^14^ ^]^ To push SSPPs further to engineering applications, a conformal SSPP waveguide constructed by ultrathin comb‐shaped metallic pattern printed on electrically thin and flexible substrates was proposed.^[^ ^15^ ^]^ Following studies demonstrated that the SSPP signals can be transmitted in a highly dense network with the crosstalk between channels suppressed effectively.^[^ ^16^, ^17^, ^18^ ^]^ These findings reveal the potential of SSPP interconnect as a promising solution for data transfer, and pave the way for SSPP applications in the next‐generation communication systems.^[^ ^19^, ^20^ ^]^

In communication systems, the transmitting information signal may suffer from large path losses for medium‐ and long‐distance communications. To reduce the path loss, a power amplifier is a critical component that boosts the power level of the signal to be transmitted up to the required value.^[^ ^21^ ^]^ However, the traditional RF/microwave amplifiers that reply on nonlinear amplifier chips may suffer from gain distortion and phase distortion (or delay distortion), which lead to the corruption of amplitude information and make the output phase variation against the input power.^[^ ^22^, ^23^ ^]^ Moreover, the time delay between the output and input signals will increase progressively with frequency in the bandwidth of the amplifier. Hence, applying the nonlinear amplifier chip to amplify the SSPP signals will bring even severe phase distortions, as SSPP has a larger wavenumber (or smaller wavelength) as compared to the free‐space wave at the same frequency.^[^ ^24^ ^]^

Another approach is to use parametric amplification, where the gain is obtained from the nonlinear interaction between the signal and the pump without using amplifier chips.^[^ ^25^, ^26^, ^27^ ^]^ In this process, the energy is transferred from a high‐intensity pump beam to a lower intensity signal beam, thus resulting in an amplification of the signal. During this interaction, a third beam (called as idler beam) is generated, and the pump energy is fully converted to the energy of signal and idler beams without causing any heat. In particular, in the nondegenerate case where the signal and idler beams are separated, the amplification is phase insensitive and will not cause the phase distortion of the signal wave. Parametric amplification has been widely used at optical frequencies, particularly applied in laser light sources. It can also be exploited as the candidate for replacing the existing fiber amplifiers.^[^ ^28^, ^29^, ^45^ ^]^ Optical fiber parametric amplifiers can offer broadband amplification in a spectrum not accessible by other optical amplification technologies, boosting the ability of optical communication systems beyond their current limits.^[^ ^30^, ^31^, ^32^, ^33^, ^34^, ^35^ ^]^ However, as the third‐order Kerr nonlinearity of the optical fiber is weak, it normally requires a substantial number of transmission wavelengths (at the order of 10^8^ wavelengths) to achieve sufficient gain at the normal incident pump power. The optical fibers cannot realize amplification in a small number of wavelengths unless the incident pump intensity is as high as 1 × 10^18^ W m^−2^ (see Supporting Information for details).

Here, we propose to realize the parametric amplification in an SSPP system to tackle the problem of phase distortion, that is, a long‐standing problem inevitable for traditional MOS‐based power amplifiers. To make our approach suitable for on‐chip integration, the SSPP parametric amplifier must provide sufficient gain within a short propagation distance. However, how to achieve this goal at low‐frequency regions (e.g., microwave and below) is an extremely challenging task. With this question in mind, we first theoretically investigate the parametric amplification in different platforms, that is, optical SPP waveguide^[^ ^36^ ^]^ and SSPP waveguide. The theoretical calculations reveal that to achieve sufficient parametric gain at only a small number of SSPP wavelengths (*λ*), the system must satisfy the following conditions simultaneously: 1) nonlinear material with high Kerr nonlinearity, 2) minimized Ohmic loss, and 3) extreme confinement of electromagnetic fields. SSPPs have the unique properties of low loss, strong field confinement, and great flexibility in manipulating the dispersion behavior.^[^ ^37^, ^38^, ^39^, ^40^ ^]^ Our theoretical model predicts that, in an SSPP waveguide, the parametric gain up to 9.14 dB can be achieved in a short nonlinear propagation length (only 6*λ*). Experiments are performed to study the parametric amplification in SSPP waveguides with different nonlinear propagation distances and the measured results show excellent agreements with our theoretical predictions. Phase‐matching conditions can be flexibly tuned by varying the bias voltage, allowing for multi‐frequency parametrical amplification of SSPP signals. Particularly, we observe that the plasmonic parametric amplifier can significantly suppress the phase distortion of the amplified SSPP signals, in sharp contrast to MOS‐based amplifier chips which suffer from serious signal distortions.

## 2 Theory and Analysis

To start with, we investigate the key factors that will affect the parametric gain. Assuming that the light is propagating along the *z*‐direction, i.e., $E_{p}\left(z\right)=A_{p}\left(z\right)e^{ik_{p}z}$, $E_{s}\left(z\right)=A_{s}\left(z\right)e^{ik_{s}z}$, and $E_{i}\left(z\right)=A_{i}\left(z\right)e^{ik_{i}z}$, corresponding to the electric fields at the pump, signal, and idler frequencies, respectively, where *A_p_*, *A_s_*, and *A_i_* are the electric field amplitudes of the pump (*ω_p_*), signal (*ω_s_*), and idler (*ω_i_*) waves, respectively. Owing to finite dissipation losses, the complex propagation constants of the pump, signal, and idler waves acquire complex values $k_{p}=k_{p}^{\prime }+ik_{p}^{\prime \prime },k_{s}=k_{s}^{\prime }+ik_{s}^{\prime \prime }$, and $k_{i}=k_{i}^{\prime }+ik_{i}^{\prime \prime }$, where $k_{p}^{\prime },k_{s}^{\prime }$ and $k_{i}^{\prime }$ determine the propagation phases, while $k_{p}^{\prime \prime },k_{s}^{\prime \prime }$, and $k_{i}^{\prime \prime }$ determine the propagation losses. For the three‐wave mixing process in the undepleted pump regime, the signal‐wave amplitude satisfies the following differential equation

(1) $$\begin{array}{c} \frac{d^{2}A_{s}}{dz^{2}}+\left(i\Delta k^{\prime }+\Delta k_{1}^{\prime \prime }\right)\frac{dA_{s}}{dz}-\frac{\omega_{s}^{2}\omega_{i}^{2}{\left(\chi_{\mathrm{eff}}^{\left(2\right)}\right)}^{2}{A_{p}}^{2}}{k_{s}k_{i}^{*}c_{0}^{4}}e^{-\left(\Delta {k^{\prime \prime }}_{2}+\Delta {k^{\prime \prime }}_{1}\right)z}A_{s}=0 \end{array}$$

where $\Delta k_{1}^{\prime \prime }=k_{p}^{\prime \prime }+k_{i}^{\prime \prime }-k_{s}^{\prime \prime }$ and $\Delta k_{2}^{\prime \prime }=k_{p}^{\prime \prime }-k_{i}^{\prime \prime }+k_{s}^{\prime \prime }$ denote the attenuation mismatch between the pump, signal, and idler waves, *c*_0_ and $\chi_{\mathrm{eff}}^{\left(2\right)}$ stand for the light velocity and the second‐order nonlinear susceptibility, respectively, and * represents the complex conjugation. The phase mismatch of the parametric process is given by $\Delta k^{\prime }=k_{s}^{\prime }+k_{i}^{\prime }-k_{p}^{\prime }$.

The general solution to Equation (1) is:

(2) $$A_{s}\left(z\right)=e^{-\frac{a_{1}z}{2}}\left(C_{1}I_{n}\left(\frac{2\sqrt{b_{1}{e^{-}}^{c_{1}z}}}{c_{1}}\right)+C_{2}K_{n}\left(\frac{2\sqrt{b_{1}{e^{-}}^{c_{1}z}}}{c_{1}}\right)\right)$$

where $a_{1}=i\Delta k^{\prime }+\Delta k_{1}^{\prime \prime }$, $b_{1}=\frac{\omega_{s}^{2}\omega_{i}^{2}{\left(\chi_{\mathrm{eff}}^{\left(2\right)}\right)}^{2}A_{p}^{2}}{\left(k_{s}^{\prime }+ik_{s}^{\prime \prime }\right)\left(k_{i}^{\prime }-ik_{i}^{\prime \prime }\right)c_{0}^{4}}$, $c_{1}=\Delta_{1}^{\prime \prime }+\Delta_{2}^{\prime \prime }$, *n* = *a*_1_ */c*_1_, *I_n_* ( · ) and *K_n_* ( · ) respectively denote the *n*‐th order modified Bessel functions of the first and second kinds, respectively. *C*_1_ and *C*_2_ are constants to be determined by the initial values of signal and idler wave (see Section S1, Supporting Information). The signal gain varying with the nonlinear waveguide length *L* is derived as:

(3) $$G={\left|\left[\frac{\left(\frac{a_{1}}{2}I_{n}\left(t\right)+\sqrt{b_{1}}{I^{\prime }}_{n}\left(t\right)\right)K_{n}\left(t\sqrt{e^{-c_{1}L}}\right)-\left(\frac{a_{1}}{2}K_{n}\left(t\right)+\sqrt{b_{1}}{K^{\prime }}_{n}\left(t\right)\right)I_{n}\left(t\sqrt{e^{-c_{1}L}}\right)}{\sqrt{b_{1}}\left[I_{n}^{\prime }\left(t\right)K_{n}\left(t\right)-I_{n}\left(t\right)K_{n}^{\prime }\left(t\right)\right]}\right]\right|}^{2}{e^{-\left(a_{1}+2k_{s}^{\prime \prime }\right)}}^{L}$$

where we have introduced a constant $t=2\sqrt{b_{1}}/c_{1}$. The detailed mathematical derivations are given in Supplementary Information. Note that the parametric amplification through a four‐wave mixing process can be treated similarly and the signal gain still takes the form of Equation (3) with *a*_1_, *b*_1_, and *c*_1_ updated as Equation (S8), Supporting Information given the supporting information. Our calculations indicate that, for a given nonlinear propagation length, the signal gain mainly depends on the nonlinear susceptibility, the incident pump intensity, and the waveguide loss. In the absence of losses, the signal gain monotonically increases as the propagation length increases. On the contrary, the presence of finite dissipation losses always sets an ultimate limit of the maximum signal gain we can achieve.

**Figure 1:**
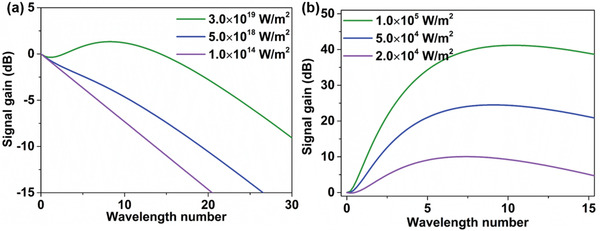
Signal gains of different waveguides at different pump intensities. a) The optical SPP. b) The spoof plasmonic waveguide. Note that those parameters used to calculate the signal gains of the optical SPP are taken from Ref. [30].

To illustrate this point and demonstrate the unique properties of spoof surface plasom polaritons (SSPPs) in parametric amplification, we compare the signal gains of two different platforms (i.e., metal SPPs at visible, and SSPPs at microwave) using the theoretical model above. As shown in **Figure** **1**a, since the optical SPPs (Au/Porous silicon) suffer from high Drude losses, the corresponding signal wave cannot be amplified unless the pump light intensity reaches as high as 3 × 10^19^ W m^−2^, which has to be generated by a femtosecond laser. To circumvent this limitation, an SSPP parametric amplifier with small loss and high field confinement simultaneously is investigated. Here we adopt the typical ultrathin comb‐shaped SSPP structure^[^ ^9^ ^]^ loaded with nonlinear varactors, in which the detailed geometrical parameters will be given in the next section. As illustrated in Figure 1b, compared with the optical SPP, the SSPP waveguide can generate greater peak gain at smaller pump intensity. For example, under the incident pump intensity of 1 × 10^5^ W m^−2^ (corresponding to 28.72 dBm in a typical comb‐shaped SSPP waveguide), the parametric gain up to 40 dB can be achieved in a very short nonlinear propagation length (10*λ*). These results indicate that the SSPP parametric amplification can realize a high gain in an ultra‐compact device, opening up the possibilities for on‐chip integration in the integrated circuits.

## 3 Design and Experimental Results

**Figure 2:**
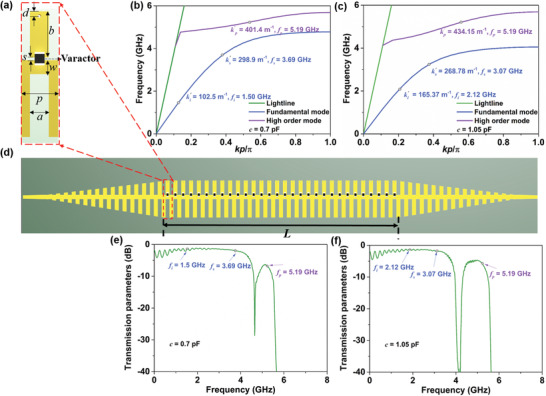
a) Unit structure of the spoof plasmonic waveguide loaded with the varactor diode, where *a* = 2 mm, *b* = 4.95 mm, *d* = 0.5mm, *p* = 4 mm, *w* = 1.5 mm, and *s* = 0.3 mm. b) The simulated dispersion relation with capacitance *c* = 0.7 pF. The condition for phase matching is $k_{p}^{\prime }=401.4$ m^−1^ at *f_p_* = 5.19 GHz, $k_{s}^{\prime }=298.9$ m^−1^ at *f_s_* = 3.69 GHz, and $k_{i}^{\prime }=102.5$ m^−1^ at *f_i_* = 1.50 GHz. c) The simulated dispersion relation with capacitance *c* = 1.05 pF. The phase‐matching condition is satisfied when $k_{p}^{\prime }=434.15$ m^−1^ at *f_p_* = 5.19 GHz, $k_{s}^{\prime }=268.78$ m^−1^ at *f_s_* = 3.07 GHz, and $k_{i}^{\prime }=165.37$ m^−1^ at *f_i_* = 2.12 GHz. d) spoof plasmonic parametric amplifier with the nonlinear waveguide length *L*. The three‐wave mixing process involves the conversion of pump wave to signal wave and idler wave. By suitable design of the waveguide structure, the phase‐matching condition is satisfied and the signal amplification occurs. e, f) are simulated transmission parameters of the spoof plasmonic waveguide at different capacitances.

The spoof plasmonic unit structure loaded with a varactor is shown in **Figure** **2**a, where the structural parameters are *a* = 2 mm, *b* = 4.95 mm, *d* = 0.5 mm, *p* = 4 mm, *s* = 0.3 mm, and *w* = 1.5 mm. The dielectric substrate is Rogers 4003C with a dielectric constant of 3.55, tangent loss of tan*δ* = 0.0027, and thickness of 0.508 mm. The thickness of metal copper is 0.018 mm. At microwave frequencies, the lacking of nonlinear materials can be overcome by introducing the varactor, whose capacitance varies with applied voltage.^[^ ^41^ ^]^ In terms of the phase‐matching condition of the second harmonic generation,^[^ ^38^, ^42^ ^]^ when nonlinear mixing occurs in a waveguide instead of a bulk medium, the phase‐matching condition for three‐wave mixing is possible to be satisfied by designing the dispersion characteristics of different SSPP modes in the waveguide.

We investigate the linear dispersion behaviors through simulations using the Eigen‐mode solvers, as sketched in Figure 2b,c, which display the relationships among the pump, signal, and idler wavenumbers of SSPPs. Note that the dispersion curves for the fundamental and high‐order modes gradually deviate from the light‐line, and then asymptotically approach different cutoff frequencies. Owing to the phase‐matching limitation, the wavenumber of the pump wave must be larger than that of the signal and idler waves. As depicted in Figure 2b, when the capacitance *c* = 0.7 pF, the dispersion behaviors of high‐order mode and fundamental mode display the pump, signal, and idler phase constants and frequencies, respectively, where $k_{p}^{\prime }=k_{s}^{\prime }+k_{i}^{\prime }$ and *f_p_* = *f_s_* + *f_i_*. When the capacitance is switched to 1.05 pF and the corresponding pump frequency remains unchanged, different phase matching is also realized (see Figure 2c). Thus, at a constant pump frequency, varied capacitances of the loaded varactor give rise to the variation of dispersion behaviors, allowing for the phase‐matching condition to be satisfied for multiple frequencies of the amplified signals.

High‐efficiency transmission of a waveguide at the corresponding phase‐matching point is essential to enhance the conversion efficiency of nonlinear processes. An SSPP waveguide consisting of 30‐unit cells is designed, as shown in Figure 2d, where its nonlinear length is *L* = 120 mm. To improve the linear transmission effect, transition sections with a length of 72 mm are introduced at both ends of the SSPP waveguide. From Figure 2e,f, we observe that high‐efficiency transmissions at different capacitances are verified by the simulation. In addition, the transmission of the pump wave is slightly lower than that of the signal and idler waves, but it is acceptable for engineering applications.

**Figure 3:**
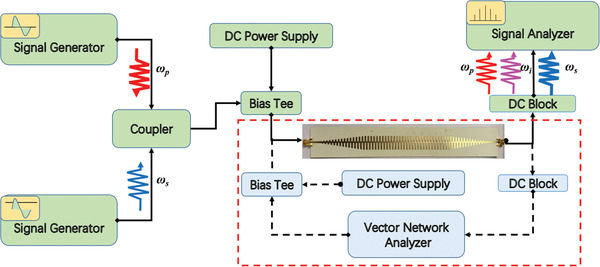
Diagram of experimental setup employed to measure the transmission parameters and scattered spectral power distributions of the parametric amplification. The linear transmission parameters setup is shown in the red dotted‐line block diagram.

Considering the influence of the varactor's parasitic resistance on the transmission performance, we fabricate the spoof plasmonic waveguide loaded with the varactors (MAVR‐000120‐1141) and build the test platform (see the red dotted‐line block diagram of **Figure** **3**). When the parasitic resistance is about 3 ohms, experimental measurement and software simulation have demonstrated that the waveguide has a high transmission efficiency (see Section S3, Supporting Information). As a result, the incident pump and signal waves can be efficiently coupled into the nonlinear spoof plasmonic waveguide, providing a good precondition to achieve high‐efficiency parametric amplification.

Figure 3 present a comprehensive setup of nonlinear experiments to observe the SSPP signal amplification in the proposed spoof plasmonic waveguide. Both the incident pump and lower‐frequency signal wave generated from different signal generators are injected into the spoof plasmonic waveguide by a coupler, and then the signal spectrum can be collected by the signal analyzer (see the Section 5).

**Figure 4:**
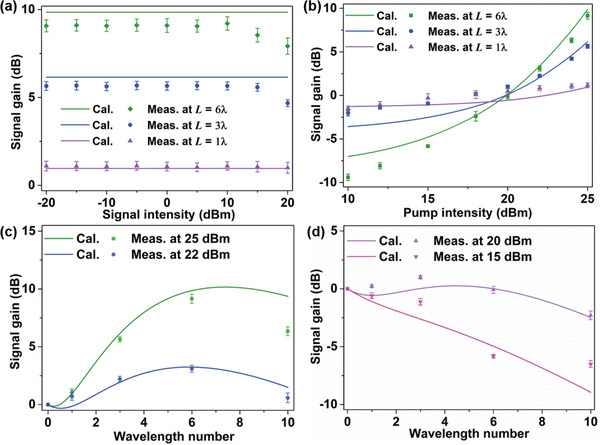
Calculated and measured signal gains of the parametric amplifier. a) Measured signal gains of different nonlinear lengths with the incident signal intensity varied from ‐20 to 20 dBm, in which the pump intensity remains 25 dBm. b) Measured and calculated signal gains of different nonlinear lengths with the incident pump intensity varied from 10 to 25 dBm, in which the signal intensity is 10 dBm. c) Signal gains varied with the wavelength number at the pump intensities of 25 and 22 dBm. d) Signal gains varied with the wavelength number at the pump intensities of 20 and 15 dBm. Here, the applied bias voltage of the waveguide is 1.73 V, and the pump and signal frequencies are 5.15 and 3.69 GHz, respectively. Note that the error bars in all figures indicate the system uncertainly in the respective measurements and have been calculated by taking the standard deviations from seven measurements.

Take the applied bias voltage of 1.73 V as an example, corresponding to the capacitance of 0.7 pF. When the pump and signal frequencies are 5.15 and 3.69 GHz, the phase‐matching condition of parametric amplification is satisfied (details in Section S4, Supporting Information). The collected nonlinear spectrum under a constant pump intensity (25 dBm) at a series of signal intensities varying from −20 to 20 dBm is sketched in **Figure** **4**a. When the nonlinear length is 120 mm, we observe that the signal gain remains constant when the initial signal intensity is below 10 dBm, and gradually diminishes as the signal intensity increases. However, when the nonlinear waveguide length decreases from 6*λ* (120 mm) to 1*λ* (20 mm), the corresponding signal gain tends asymptotically to stabilize at the whole signal intensity range. In contrast to the measured results, the theoretical calculations obtained by Equation (3) are independent of the initial signal intensity. This discrepancy is because that the theoretical model does not consider the pump depletion, which is not negligible when the signal gain is large enough.

Based on the above analysis, the incident signal intensity is fixed at 10 dBm in the following measurements. The signal gain with the pump intensity varying from 10 to 25 dBm is shown in Figure 4b. We observe that the amplified gain monotonically increases with the incident pump intensity. There exist some discrepancies between the calculated and measured results due to the errors of 1) unavoidable manufacturing error, 2) welding technology, and 3) instrument and environmental noises. Nevertheless, we still observe good agreement between these results.

Figure 4c,d exhibit the collected nonlinear spectra under different constant pump intensities at a series of nonlinear waveguide lengths varying from 10*λ* (200 mm) to 1*λ* (20 mm). Owing to the loss of the proposed waveguide, the signal gain shows a trend of increasing first and decreasing next as the nonlinear length increases, as displayed in Figure 4c, agreeing remarkably well with the theoretical prediction. Moreover, when the incident pump intensity gradually descends and reaches a certain constant, the signal wave cannot be amplified and the generated gain is below the waveguide loss (Figure 4d). Note that for different incident pump intensities, the corresponding peak gains are achieved from different waveguide lengths. Thus, the theoretical predictions provide useful guidance for us to choose the waveguide length in accordance with the requirement of practical applications.

**Figure 5:**
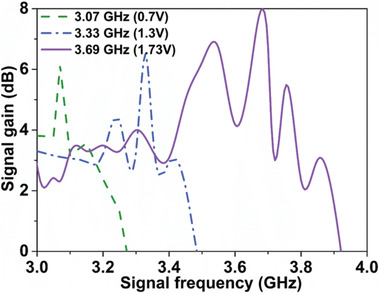
Signal gains varied with signal frequencies under different phase‐matching conditions by applying different bias voltages. Note that the incident pump frequency and intensity are 5.15 GHz and 25 dBm, respectively, the incident signal intensity is 20 dBm, and the nonlinear length of the spoof plasmonic waveguide is 120 mm.

Additionally, multi‐frequency parametrically amplified SSPP signals can be obtained by applying bias voltages to the varactors loaded on spoof plasmonic waveguide according to the analyses of dispersion behaviors (see Figure 2). In the following measurements, the length of the spoof plasmonic waveguide remains 120 mm. The incident pump frequency and intensity are 5.15 GHz and 25 dBm, respectively, and the incident signal intensity is a constant of 20 dBm. When the applied bias voltage is 0.7 V, the realization of phase matching is evidenced by the sharp peak in the conversion efficiency at the signal wave of 3.07 GHz, as illustrated in **Figure** **5**. When the applied bias voltage switches to 1.3 and 1.73 V, the peak gains occur at the signal waves of 3.33 and 3.69 GHz, respectively. We also observe that the signal gains at different phase‐matching points are different from the transmission characteristics of the nonlinear spoof plasmonic waveguide.

**Figure 6:**
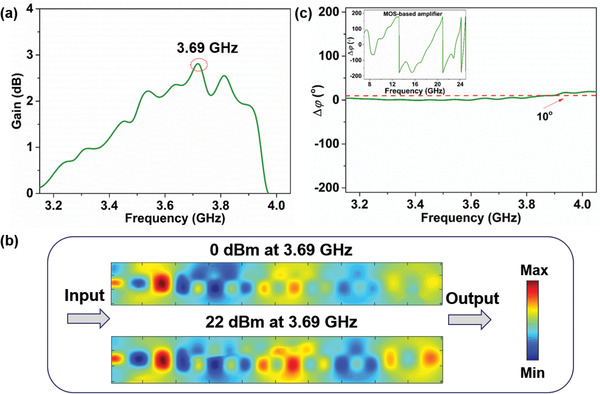
The performance comparison between the parametric amplifier and the MOS‐based amplifier. a) Measured signal gains of the spoof plasmonic parametric amplifier by the signal generator and vector network analyzer. b) Near‐field distributions at 3.69 GHz under the pump intensities of 0 and 22 dBm. c) Phases varied with the working frequency based on the spoof plasmonic parametric amplifier. The inset shows phase distortion of the signal wave for spoof plasmonic waveguide loaded with amplifier chip. Note that the simulated parameters have been listed in Ref. [24].

In wireless communication systems, the signal distortion will damage the signal integrity for high‐speed digital designs.^[^ ^43^ ^]^ To compare the performance of amplifiers based on the parametric amplification and amplifier chip, we investigate the phase differences between the amplified and original signals in different spoof plasmonic waveguides. First, for the spoof plasmonic parametric amplifier, we must ensure that the analyzed signal waves are in the amplification region. Most notably, we remark that the measurement setup of its phase difference is not the same as that of the scattered power spectrum (see Section S5, Supporting Information). As illustrated in **Figure** **6**a, the signal wave can be amplified when the incident pump frequency and intensity are 5.15 GHz and 22 dBm, respectively. To observe the signal amplification phenomena more intuitively, we measure the near‐field distributions at 3.69 GHz under the pump intensities of 0 dBm and 22 dBm (details in Section S5, Supporting Information), as shown in Figure 6b. Additionally, the corresponding 1D plot electric field diagram is shown in Figure S5, Supporting Information. Compared to the near field generated from the pump intensity of 0 dBm, the near field generated from the pump intensity of 22 dBm is notably enhanced at the output port when the signal wave propagates along the SSPP‐based waveguide. It is also clearly seen that the phase at 3.69 GHz remains almost unchanged at different pump intensities.

Meanwhile, the phases varied with the operating frequency from 3.2 to 4.0 GHz are also measured by the signal generator and vector network analyzer, as shown in Figure 6c. The phase change induced by the spoof plasmonic parametric amplifier remains about 5° in the working frequency band. Then, for a MOS‐based amplifier, we use CST simulations to obtain the phase relation between double‐side plasmonic waveguides with and without the amplifier chip. As shown in the inset of Figure 6c, the phase change with the operating frequency (Δ*φ*) undergoes serious distortions, causing the signal integrity problem. In particular, the corresponding transmission parameters are shown in Figure S6, Supporting Information. By comparison, the plasmonic parametric amplifier has a stable response and negligible signal distortion.

## 4 Conclusion

We demonstrated a high‐performance and reconfigurable parametric amplifier for SSPP signals, and both theoretical analysis and experimental results confirmed that the signals can be amplified in a short propagation distance. The plasmonic amplifier is realized on an ultrathin planar platform, holding promising for on‐chip integration with microwave circuits. Owing to the flexibility of SSPP dispersive behaviors, the parametrically amplified SSPP signals at multiple frequencies were achieved with different bias voltages. More importantly, different from the scenario in MOS‐based amplifiers, the proposed plasmonic parametric amplification does not cause phase distortions. We believe that this work will expand the scope of plasmonic metamaterials and nonlinear metamaterials, and establish a foundation for novel wireless communication engineering to facilitate a fast and reliable data transfer process with advanced signal integrity.

## 5 Experimental Section

Different measurement setups were carried out to obtain the linear transmission parameters and collect the scattered power spectra of the signal waves. The vector network analyzer (Agilent N5230C) was used to measure S‐parameters at different bias voltages. DC block was introduced to prevent the DC signal from entering the device. To measure the signal amplification of the designed sample, the pump and signal intensities created from two signal generators (Agilent E8267D and E8257D) were merged using an RF coupler (Midwest Microwave, CPL‐5231‐16‐001‐79) into a single signal to excite the nonlinear SSPP waveguide. The output signal intensities were collected by the signal analyzer (Agilent N9010A). A bias Tee was placed between the coupler and the sample to feed the RF signal and DC bias into the sample simultaneously.

## Conflict of Interest

The authors declare no conflict of interest.

## Supporting information

Supporting Information

## Data Availability

Research data are not shared.
