# Supplementary material for: Reconfigurable Parametric Amplifications of Spoof Surface Plasmons
Source: Adv Sci (Weinh). 2021 Jul 4;8(17):2100795. doi: 10.1002/advs.202100795 (PMC8425943; doi:10.1002/advs.202100795)
Supplement: Supplementary file 1 — Supporting Information [file ADVS-8-2100795-s001.pdf]

© 2021 Wiley-VCH GmbH

ADVANCED SCIENCE
Open Access

# Supporting Information

for *Adv. Sci.,* DOI: 10.1002/advs.202100795

# Reconfigurable Parametric Amplifications of Spoof Surface Plasmons

*Xinxin Gao, Jingjing Zhang,* Yu Luo,* Qian Ma, Guo Dong Bai, Hao Chi Zhang, and Tie Jun Cui**

# Supplementary Information for

# Reconfigurable parametric amplifications of spoof surface plasmons

*Xinxin Gao*[1,2,4]*, Jingjing Zhang*[1,2,4,*]*, Yu Luo*[3,*]*, Qian Ma*[1,2,4]*, Guo Dong Bai*[1,2]*, Hao Chi Zhang*[1,2,4]*, and Tie Jun Cui*[1,2,4,*]

[1] *Institute of Electromagnetic Space, Southeast University, Nanjing 210096, China*
[2] *State Key Laboratory of Millimeter Waves, Southeast University, Nanjing 210096, China*
[3] *School of Electrical and Electronic Engineering, Nanyang Technological University, Nanyang Avenue 639798, Singapore*
[4] *Center of Intelligent Metamaterials, Pazhou Laboratory, Guangzhou 510330, China*

[*] *Corresponding authors: E-mail: zhangjingjing@seu.edu.cn; luoyu@ntu.edu.sg; and tjcui@seu.edu.cn*

**This file includes:**

1. Theoretical analysis
2. Signal gain of the optical fiber at different pump intensity
3. Transmission performance of spoof plasmonic waveguide
4. Determination of phase-matching points
5. Experimental setup to measure the signal phase and near-field distributions
6. The 1D plot of the electric field
7. Simulated signal gain of a MOS-based SSPP amplifier

Supplementary Figures S1-S6

Supplementary References 1-7.

## 1. Theoretical analysis

When the light is propagating along the *z*-direction, we have

$$E_s(z)=A_s(z)e^{ik_s z}=A_s(z)e^{ik_s' z-k_s'' z},\quad E_i(z)=A_i(z)e^{ik_i z}=A_i(z)e^{ik_i' z-k_i'' z},$$

$$E_p(z)=A_p(z)e^{ik_p z}=A_p(z)e^{ik_p' z-k_p'' z},$$

which correspond to the electric fields of the signal, idler, and pump waves, respectively. Here, $A_p$, $A_s$, and $A_i$ are electric field amplitudes of the pump ($\omega_p$), signal ($\omega_s$), and idler ($\omega_i$) waves, respectively, satisfying the following relations:

$$\frac{dA_s(z)}{dz}=\frac{i\omega_s^2}{k_s c_0^2}\chi_{\mathrm{eff}}^{(2)}A_p(z)A_i^*(z)e^{-i\Delta k' z}e^{-\Delta k_1'' z} \quad \text{(S1a)}$$

$$\frac{dA_i(z)}{dz}=\frac{i\omega_i^2}{k_i c_0^2}\chi_{\mathrm{eff}}^{(2)}A_p(z)A_s^*(z)e^{-i\Delta k' z}e^{-\Delta k_2'' z} \quad \text{(S1b)}$$

where $\Delta k_1'' = k_p'' + k_i'' - k_s''$ and $k_2'' = k_p'' - k_i'' + k_s''$ denote the attenuation mismatch between the pump, signal, and idler waves, $c_0$ and $\chi_{\mathrm{eff}}^{(2)}$ stand for the light velocity and the second-order nonlinear susceptibility, and $*$ represents the complex conjugation. The phase mismatch of the parametric process is given by $\Delta k' = k_s' + k_i' - k_p'$. For the three-wave mixing process in the undepleted pump regime, the signal wave amplitude satisfies the following differential equation

$$\frac{d^2A_s}{dz^2}+\left(i\Delta k'+\Delta k_1''\right)\frac{dA_s}{dz}-\frac{\omega_s^2\omega_i^2\left(\chi_{\mathrm{eff}}^{(2)}\right)^2 A_p^{\ 2}}{k_s k_i^* c_0^4}e^{-\left(\Delta k_2''+\Delta k_1''\right)z}A_s=0 \quad \text{(S2a)}$$

$$\frac{d^2A_s}{dz^2}+a_1\frac{dA_s}{dz}-b_1 e^{-c_1 z}A_s=0 \quad \text{(S2b)}$$

where $a_1 = i\Delta k' + \Delta k_1''$, $b_1=\dfrac{\omega_s^2\omega_i^2\left(\chi_{\mathrm{eff}}^{(2)}\right)^2 A_p^2}{\left(k_s'+ik_s''\right)\left(k_i'-ik_i''\right)c_0^4}$, $c_1 = \Delta k_1'' + \Delta k_2''$. The general solution to Equation (S2) is

$$A_s(z)=e^{-\frac{a_1 z}{2}}\left(C_1 I_n\left(\frac{2\sqrt{b_1 e^{-c_1 z}}}{c_1}\right)+C_2 K_n\left(\frac{2\sqrt{b_1 e^{-c_1 z}}}{c_1}\right)\right) \tag{S3}$$

where $n = a_1/c_1$, $I_n(\cdot)$ and $K_n(\cdot)$ denote the $n$-th order modified Bessel functions of the first and second kinds, respectively; and $C_1$ and $C_2$ are constants to be determined by the initial values of signal and idler waves:

$$A_s(z)\big|_{z=0}=A_s(0) \tag{S4a}$$

$$A_i^*(z)\big|_{z=0}=A_i^*(0) \tag{S4b}$$

$$\left.\frac{dA_s(z)}{dz}\right|_{z=0}=\frac{i\omega_s^2}{k_s c_0^2}\chi_{\text{eff}}^{(2)}A_p(0)A_i^*(0) \tag{S4c}$$

Substituting Eq. (S4) into (S3), we can get

$$C_1=-\frac{A_s(0)\left[\frac{a_1}{2}K_n(t)+\sqrt{b_1}K_n'(t)\right]+K_n(t)\left.\frac{dA_s(z)}{dz}\right|_{z=0}}{\sqrt{b_1}\left[K_n(t)I_n'(t)-I_n(t)K_n'(t)\right]} \tag{S5a}$$

$$C_2=\frac{A_s(0)\left[\frac{a_1}{2}I_n(t)+\sqrt{b_1}I_n'(t)\right]+I_n(t)\left.\frac{dA_s(z)}{dz}\right|_{z=0}}{\sqrt{b_1}\left[K_n(t)I_n'(t)-I_n(t)K_n'(t)\right]} \tag{S5b}$$

Here, we have introduced a constant $t=2\sqrt{b_1}/c_1$. In general, the idler intensity at $z$ = 0 is zero, and hence Equation (S3) can be rewritten as

$$A_s(z)=A_s(0)e^{-\frac{a_1 z}{2}}\left[\frac{\left(\frac{a_1}{2}I_n(t)+\sqrt{b_1}I_n'(t)\right)K_n\left(t\sqrt{e^{-c_1 z}}\right)-\left(\frac{a_1}{2}K_n(t)+\sqrt{b_1}K_n'(t)\right)I_n\left(t\sqrt{e^{-c_1 z}}\right)}{\sqrt{b_1}\left(I_n'(t)K_n(t)-I_n(t)K_n'(t)\right)}\right] \tag{S6}$$

Therefore, the signal gain varied with the nonlinear waveguide length $L$ is derived as

$$G = \left\| \left[ \frac{\left(\frac{a_1}{2} I_n(t) + \sqrt{b_1} I_n'(t)\right) K_n\left(t\sqrt{e^{-c_1 L}}\right) - \left(\frac{a_1}{2} K_n(t) + \sqrt{b_1} K_n'(t)\right) I_n\left(t\sqrt{e^{-c_1 L}}\right)}{\sqrt{b_1}\left[I_n'(t) K_n(t) - I_n(t) K_n'(t)\right]} \right] \right\|^2 e^{-(a_1 + 2k_s'')L} \quad \text{(S7)}$$

Equation (S7) indicates that, for a given nonlinear waveguide length, the signal gain mainly depends on the nonlinear susceptibility and the waveguide loss.

Additionally, the derived formula is suitable for parametric amplification with a four-wave mixing process. When two pump waves at frequency $\omega_p$ are converted to signal and idler waves at the respective frequencies $\omega_s$ and $\omega_i$ such that $2\omega_p = \omega_i + \omega_s$, this leads to amplification of the signal wave. Here, the signal gain can be rewritten as

$$G = \left\| \left[ \frac{\left(\frac{a_2}{2} I_m(t_1) + \sqrt{b_2} I_m'(t_1)\right) K_m\left(t_1\sqrt{e^{-c_2 L}}\right) - \left(\frac{a_2}{2} K_m(t_1) + \sqrt{b_2} K_m'(t_1)\right) I_m\left(t_1\sqrt{e^{-c_2 L}}\right)}{\sqrt{b_2}\left[I_m'(t_1) K_m(t_1) - I_m(t_1) K_m'(t_1)\right]} \right] \right\|^2 e^{-(a_2 + 2k_s'')L} \quad \text{(S8)}$$

where $a_2 = i\left(k_s' + k_i' - 2k_p'\right) + \left(2k_p'' + k_i'' - k_s''\right)$ , $b_2 = \frac{\omega_s^2 \omega_i^2 \left(\chi_{\text{eff}}^{(3)}\right)^2 A_p^4}{\left(k_s' + ik_s''\right)\left(k_i' - ik_i''\right) c_0^4}$ , $c_2 = 4k_p''$ ,

$t_1 = 2\sqrt{b_2}/c_2$ , and $m = a_2/c_2$ . Note that $\chi_{\text{eff}}^{(3)}$ is the third-order nonlinear susceptibility. Additionally, the power intensity is expressed by $I = P/A_{\text{eff}} \propto |A_P|^2$, where $P$ is the power and $A_{\text{eff}}$ is the effective mode area. The effective mode area, $A_{eff}$, is the ratio between the total density per unit length and the peak power density [6, 7]

$$A_{\text{eff}} = \frac{\int_{A_\infty} W(r)\, dA}{\max\{W(r)\}} \quad \text{(S9)}$$

where $W(r)$ is the power density defined as,

$$W(r) = \frac{1}{2}\text{Re}\left\{\frac{d[\omega\varepsilon(r)]}{d\omega}\right\}|E(r)|^2 + \frac{1}{2}\mu_0 |H(r)|^2 \quad \text{(S10)}$$

where $\varepsilon$ and $\mu_0$ are the permittivity and permeability, respectively. Here, the effective mode area can be calculated by calculating numerically the electric and magnetic fields of the SSPP waveguide, using a full-wave numerical method in CST Studio Suite. Besides, in our experimental measurement, the power is input from a signal generator and its unit is dBm, where dBm is a ratio based on 1mW power. Thus, dBm and W can be interconverted as

$$P_{\mathrm{dBm}} = 10\log_{10}\left(\frac{P_{\mathrm{W}}}{P_0}\right) \tag{S11}$$

where $P_0$ = 1 mW.

## 2. Signal gain of the optical fiber at different pump intensity

We use the above theoretical model to study the parametric amplification of optical fiber. Note that the parametric amplification of optical fiber is based on a four-wave mixing process. The calculated parameters are listed in Reference [1]. As shown in Figure S1a, the peak gain generated from the optical fiber can be as high as about 50 dB at the incident pump intensity of $1.5\times10^{11}$ W/m$^2$. However, the high gain is achieved at the cost of long nonlinear length (at the order of $10^8$ wavelengths) due to the small third-order Kerr nonlinearity. Figure S2b shows that the optical fiber cannot realize the signal amplification within a small number of wavelengths until the corresponding pump intensity reaches about $1\times10^{18}$ W/m$^2$.

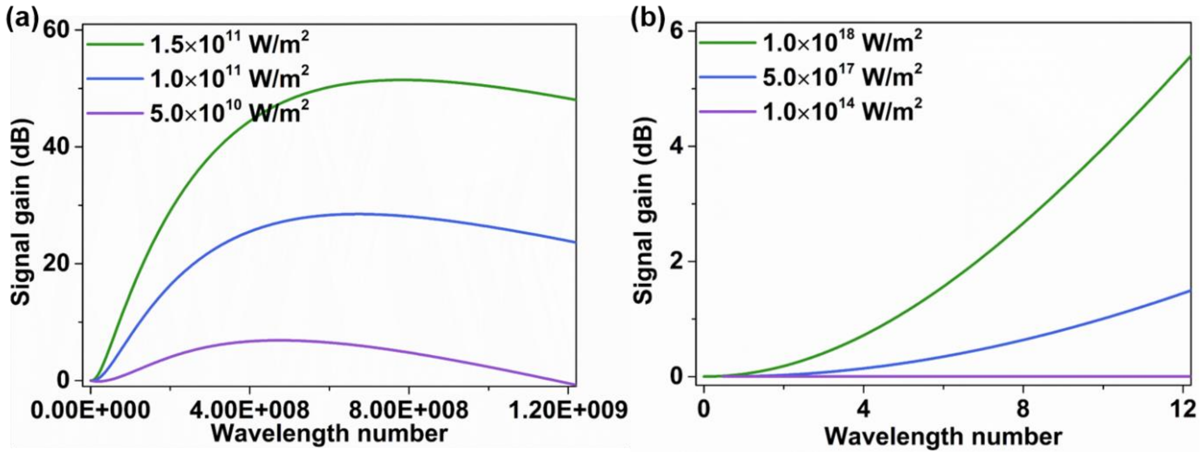


**Figure S1.** Signal gains of the optical fiber at different pump intensities.

## 3. Transmission performance of the spoof plasmonic waveguide

To verify the signal amplification in the nonlinear spoof plasmonic waveguide, we fabricate a sample loaded with 30 varactor diodes (MAVR-000120-1141). But the parasitic resistance of varactor diodes will weaken the transmission performance of the waveguide. The transmission parameters of the proposed nonlinear spoof plasmonic waveguide are measured by the vector network analyzer. The observed transmission parameters under different bias voltages ($U$ = 1.73 V and 0.7 V) agree very well with the simulated results for different capacitances ($c$ = 0.7 pF and 1.05 pF), as shown in Figure S2. Although the measurement results at high-order modes are lower than the simulations, good transmission effects of the proposed plasmonic waveguide are guaranteed in the working band, promising a good precondition to further obtain high signal amplification.

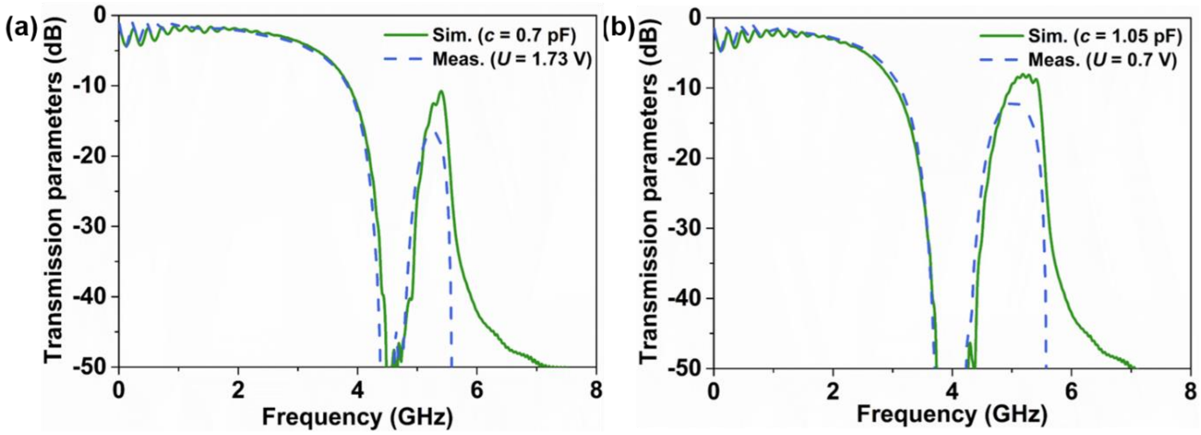


**Figure S2.** Measured transmission results of the nonlinear spoof plasmonic waveguide at different applied voltages. a) When the bias voltage is $U$ = 0.7 V, the corresponding measured results are consistent with the simulation as the capacitance is 1.05 pF. b) When the bias voltage is $U$ = 1.73 V, the corresponding measured results are consistent with the simulation as the capacitance is 0.7 pF. Note that the parasitic resistance of the varactor is 3-ohm.

## 4. Determination of the phase-matching point

Figure S3a illuminates the collected nonlinear signal spectra under a constant input signal

frequency (3.69 GHz) and external incident signal intensity (20 dBm) at a series of excitation pump frequencies with a pump intensity of 25 dBm, where the signal gain peak occurs at $f_p$ = 5.15 GHz. Besides, provided a constant pump intensity (25 dBm) and external signal intensity (20 dBm), the proposed plasmonic waveguide can identify the maximum signal gain at $f_s$ = 3.69 GHz, as shown in Figure S3c. Thus, the above phenomena designate that the phase-matching condition is satisfied when $f_p$ = 5.15 GHz and $f_s$ = 3.69 GHz. We note that there is a small frequency offset at the phase-matching points between simulation (Figure 3a) and measurement owing to the manufacturing error and the tolerance of the varactor. However, its impact on the phenomenon of signal amplification is very weak. The peak gain can achieve 7.53 dB under the incident pump intensity of 25 dBm and signal intensity of 20 dBm. The effects of phase-matching are evident as the peak of the gain is shifted significantly from the pump frequencies.

The dependencies of the idler intensity on the incident pump and signal frequencies are illustrated in Figures S3b and S3d, respectively. Two distinct signals centred at $f_p$ = 5.15 GHz and $f_s$ = 3.69 GHz are generated from two signal generators. The pump and signal intensities are maintained at a constant level (25 dBm and 20 dBm), the idler wave emerging from the coupling of the pump and signal waves through the nonlinear SSPP waveguide features a distinct peak centred at $f_i$ = 1.46 GHz, verifying the phase-matching condition again. The nonlinear signal amplification characteristics at the phase-matching condition are further illustrated in Figures S3e and S3f, showing that the idler intensity increases gradually as the signal or the pump intensity increases.

In addition, owing to the material and varactor losses, the phase and attenuation constants of the proposed spoof SPP waveguide can be calculated by the method of electromagnetic parameter retrieval. [2, 3] The propagation phases and losses at the pump, signal, and idler frequencies are $k_p'$= 108 m$^{-1}$, $k_s'$ = 307 m$^{-1}$, $k_i'$= 108 m$^{-1}$, $k_p''$ = 13.77 m$^{-1}$, $k_s''$ =

7.76 m$^{-1}$, and $k_i'' = 0.82$ m$^{-1}$, respectively, which are extremely smaller than those of the optical SPP. [4]

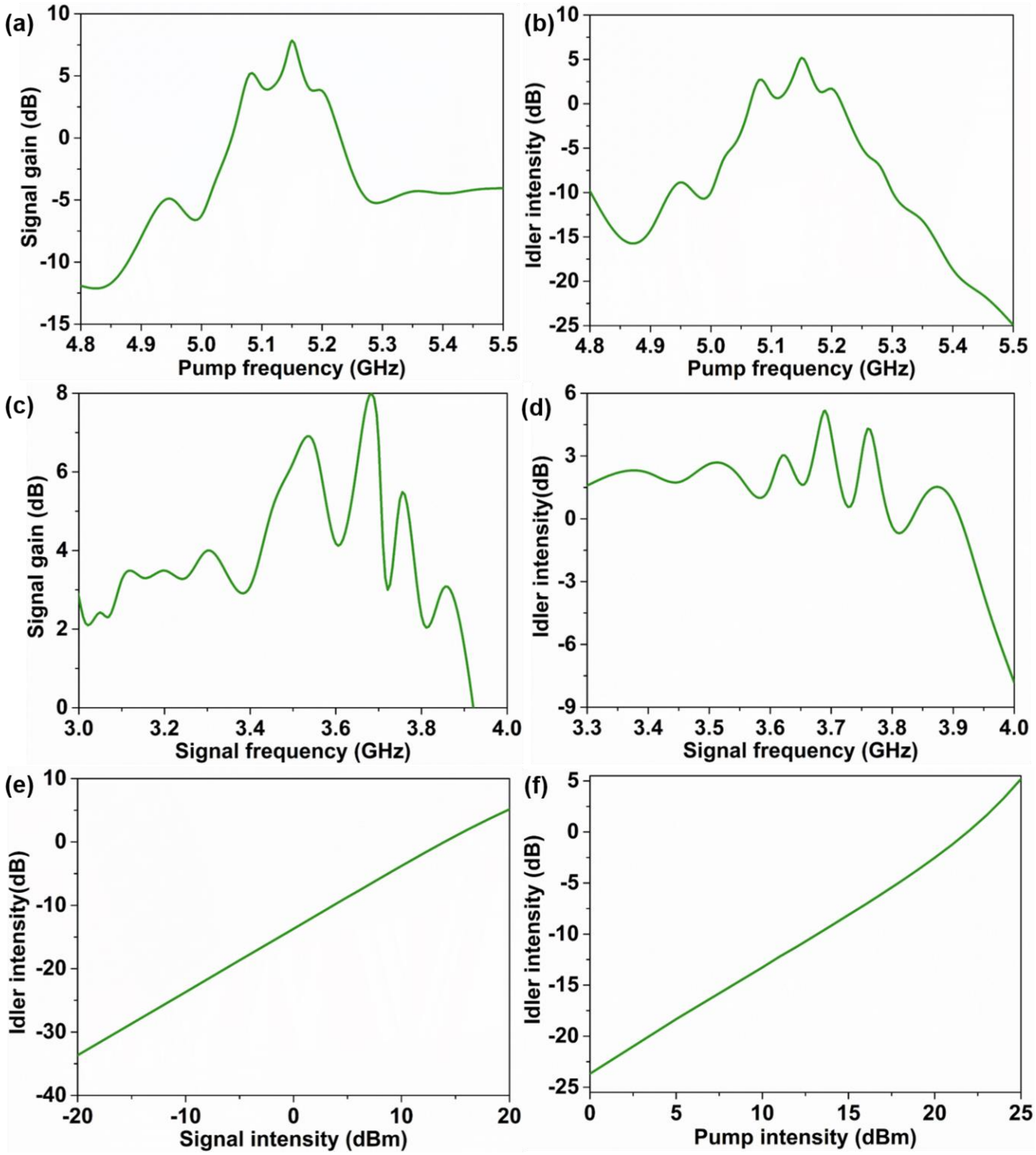


**Figure S3.** Parametric amplification performance characteristics. a) and b) The signal gains and idler intensities of the SSPP waveguide with different pump frequencies, in which the pump and signal intensities are 25 dBm and 20 dBm, respectively, and the signal frequency is 3.69 GHz. c) and d) The signal gains and idler intensities of the SSPP waveguide with different signal frequencies, in which the pump and signal intensities are 25 dBm and 20 dBm, respectively, and the signal frequency is 5.15 GHz. e)

The idler intensities with different signal intensities, in which the signal and the pump frequencies are 3.69 GHz and 5.15 GHz, respectively, and the pump intensity is 25 dBm. f) The idler intensities with different pump intensities, in which the pump and the signal frequencies are 5.15 GHz and 3.69 GHz, and the signal intensity is 20 dBm. Note that the applied bias voltage and the nonlinear length are 1.73 V and 120 mm, respectively.

## 5. Experimental setup to measure the signal phase and near field distributions

Different experimental setups are carried out to measure the transmission phase and near-field distributions of the signal wave. In the transmission phase measurement, we use the wideband signal wave and pump intensity generated from the vector network analyzer (VNA) (Agilent N5230C) and signal generator (Agilent E8267D), respectively. They are merged into an RF coupler (Midwest Microwave, CPL-5231-16-001-79) to excite the nonlinear SSPP waveguide.

To show the amplified signal more intuitively, the near-field distributions of the sample are measured. The experimental platform is set up in a microwave anechoic chamber, where a monopole probe can be automatically controlled by a stepper motor. Port 1 of VNA (Ceyear 3672B) and the signal generator (Agilent E8267D) is connected with the RF coupler. Port 2 of VNA is connected to the monopole probe to detect the near-field distributions at 3.69 GHz, where the probe is placed 2 mm above the sample. Additionally, the introduced DC block is to prevent the DC signal from entering the device and the introduced attenuator is to avoid the excessive pump intensity entering the vector network analyzer. To prevent the generated idler wave from affecting the signal waves, a high pass filter (Mini-Circuits 15542) is employed. A bias Tee is placed between the coupler and the sample to feed the RF signal and DC bias into the sample. Note that a DC-Block and 50-ohm matched load is connected to the output of the sample to suppress the DC signal and reflection.

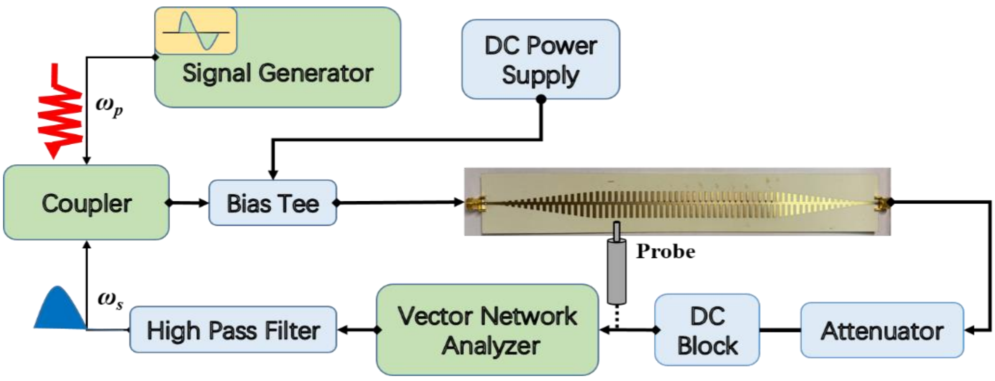


**Figure S4**. The schematic of the experimental setup for measuring the signal phase and near-field distributions at different pump intensities.

## 6. The 1D plot of the electric field

The probe scan area is shown in Figure S5a, where the vertical width (y) and the transverse length (Length) are 30 mm and 200 mm, respectively. For an SSPP-based structure, the electric fields are mainly confined around the SSPP units and the corresponding field distribution is uneven, and therefore the 1D electric field diagrams under different vertical width *y* are shown in Figure S5b to S5e. Compared to the pump intensity of 0 dBm, the pump intensity of 22 dBm can generate larger field strength when the transverse length is more than 90 mm. Thus, the proposed SSPP waveguide can realize the parametric amplification.

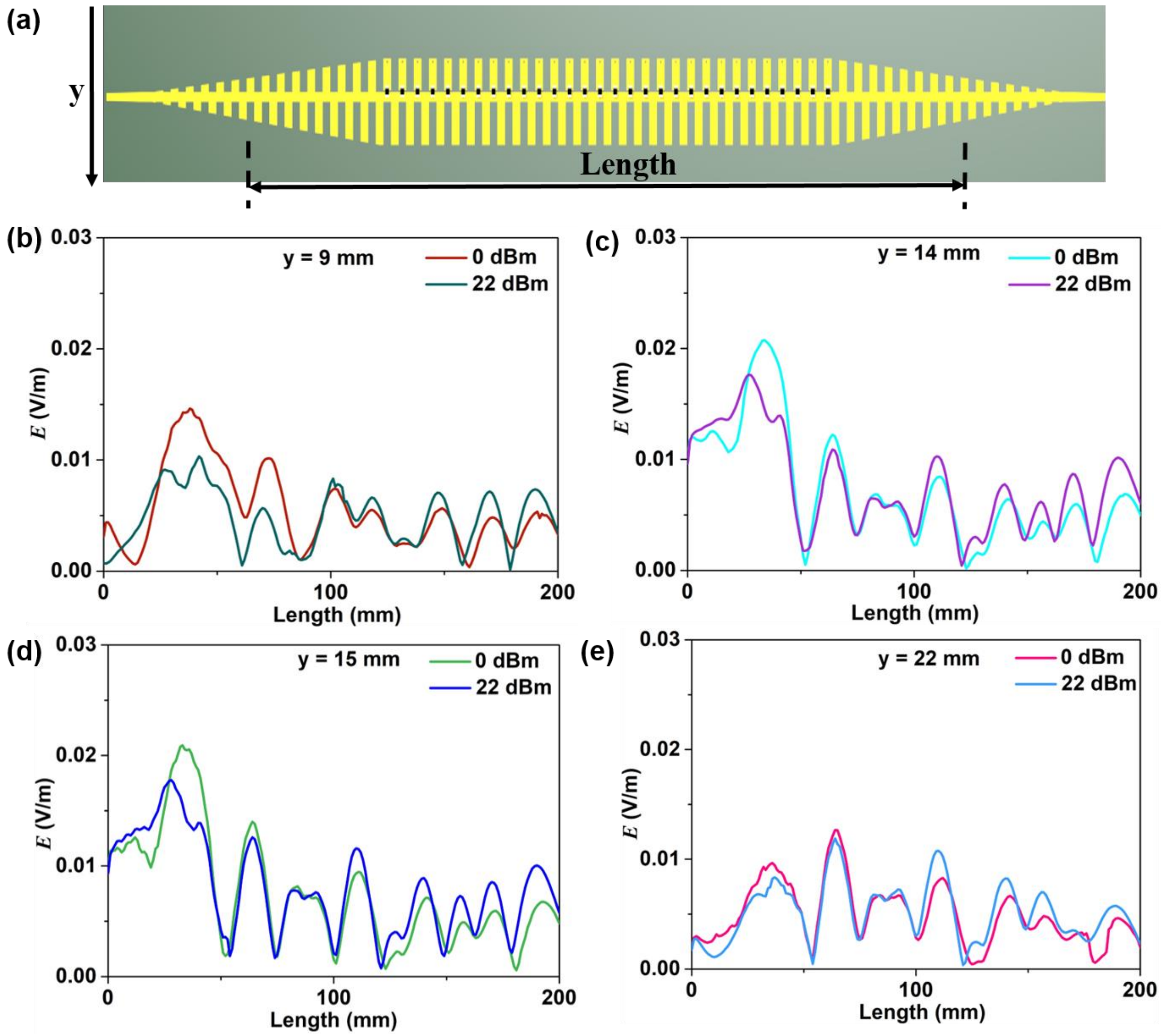


**Figure S5**. (a) SSPP waveguide with the vertical width y. (b), (c), (d), and (e) are electric field distributions under different widths of the SSPP waveguide.

## 7. Simulated signal gain of a MOS-based SSPP amplifier

To analyze the phase variable of a transistor-based SSPP amplifier, we reproduce a double-side plasmonic waveguide loaded with an amplifier chip (AMMC-6222) similar to that in Reference [5]. Details regarding the features and design method of this chip can be found in the datasheet provided by the company's website. By importing the S2P file of AMMC-6222 into Circuits and Systems modules in CST, the corresponding transmission parameters of the transistor-based amplifier can be obtained by simulations, as shown in

Figure S4, where the result fits nicely with the measured results displayed in Reference [5]. Thus, the simulation is feasible for the study of a MOS-based amplifier phase change.

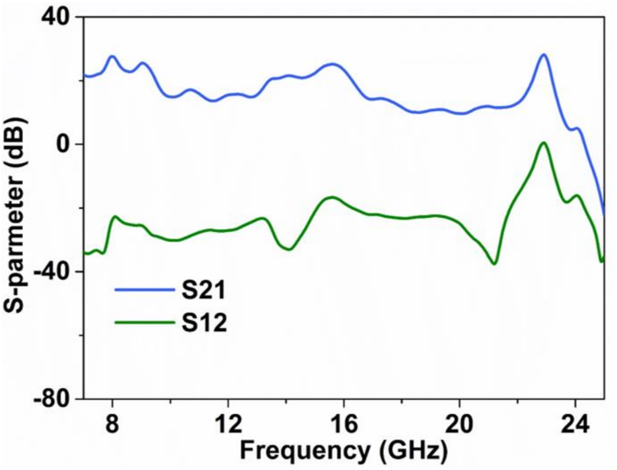


**Figure S6**. Simulated S-parameters of the double-side plasmonic waveguide mounted with amplifier chip.

## Supplementary References

[1] J. Hansryd, P. A. Andrekson, M. Westlund, J. Li, P. O. Hedekvist, *IEEE J. Sel. Topics Quantum Electron.* **2002**, 8, 506.

[2] R. Smith, D. C. Vier, T. Koschny, C. M. Soukoulis, *Phys. Rev. E* **2005**, 71, 036617.

[3] L. P. Zhang, H. C. Zhang, Z. Gao, T. J. Cui, *IEEE Trans. Antennas Propag.* **2019**, 67, 4920.

[4] A. T. Georges, *J. Opt. Soc. Am. B* **2013**, 30, 904.

[5] H. C. Zhang, S. Liu, X. P. Shen, L. H. Chen, L. M. Li, T. J. Cui, *Laser Photonics Rev.* **2015**, 9, 83.

[6] G. Agrawal, *Nonlinear Fiber Optics*, 5$^{th}$, Elsevier, Singapore **2013**.

[7] L. K. van Vugt, B. Piccione, C. H. Cho, P. Nukala, R. Agarwal, *Proc. Natl. Acad. Sci.* **2011**, 108, 10050.
